# Indian Interventional Cardiologists’ Perspectives on De-escalating Dual to Single Antiplatelet Therapy in High Bleeding Risk (HBR) Patients

**DOI:** 10.7759/cureus.91568

**Published:** 2025-09-03

**Authors:** Jagdish Chander Mohan, Saumitra Ray, Abraham Oomman, Shah VT, Baishali Nath, Anand Chitre

**Affiliations:** 1 Department of Cardiology, Jaipur Golden Hospital, New Delhi, IND; 2 Department of Invasive Cardiology, Manipal Hospital, Kolkata, IND; 3 Department of Cardiology, Apollo Hospitals, Chennai, IND; 4 Department of Cardiovascular Medicine, Surana Sethia Hospital, Mumbai, IND; 5 Department of Medical Affairs, Ajanta Pharma Limited, Mumbai, IND; 6 Department of Marketing, Ajanta Pharma Limited, Mumbai, IND

**Keywords:** antiplatelet agents, cardiovascular abnormalities, coronary artery disease, dual antiplatelet therapy, percutaneous coronary intervention

## Abstract

Background: Managing thrombotic and hemorrhagic risks associated with dual antiplatelet therapy (DAPT) in patients with a high bleeding risk (HBR) is challenging, especially in post-percutaneous coronary intervention (PCI) patients. Transitioning from DAPT to single antiplatelet therapy (SAPT) aims to reduce bleeding complications while ensuring sufficient ischemic protection. This study explored Indian interventional cardiologists’ views on de-escalation strategies in HBR patients, considering bleeding risk factors, including Indian-specific risks, such as tropical diseases and other comorbidities.

Materials and methods: A cross-sectional questionnaire-based survey was conducted from June 2024 to July 2024 among 400 interventional cardiologists in India. A structured, pre-validated questionnaire was used to gather data on the perceived prevalence of HBR in routine practice, key HBR factors specific to Indian patients (such as frailty, comorbidities, and tropical diseases), preferred SAPT agents after DAPT, perceived bleeding risk profiles of antiplatelet agents, and barriers to implementation of de-escalation. Participation was voluntary and anonymous. Responses were analyzed using descriptive statistics and reported as frequencies and proportions.

Results: Of the 375 interventional cardiologists, 193 (51.47%) reported an HBR prevalence of less than 10%, and 250 (66.67%) believed that gastrointestinal (GI) bleeding was the most common complication in patients receiving DAPT. The main HBR factors included frailty, reported by 326 (86.90%) cardiologists, chronic kidney disease (CKD) stage 3 or severe, reported by 319 (85.1%) cardiologists, and liver cirrhosis with portal hypertension, reported by 324 (86.4%) interventional cardiologists. After DAPT, 128 (34.13%) cardiologists preferred clopidogrel 75 mg, and 107 (28.53%) preferred aspirin 75 mg. Clopidogrel was seen as the least likely to cause bleeding by 179 (48.00%) interventional cardiologists, with 80 (21.33%) rating it as the best option for HBR patients de-escalating from DAPT to SAPT.

Conclusions: This study presents Indian interventional cardiologists’ perspectives on de-escalation strategies, including transitioning from DAPT to SAPT, to reduce bleeding complications, while ensuring adequate ischemic protection in patients with HBR post-PCI. Interventional cardiologists have outlined criteria for identifying critical HBR factors relevant to the Indian population. Clopidogrel was the most preferred medication for transitioning to SAPT in HBR patients. Simplified Indian bleeding risk scoring tools and tailored approaches are essential for improving DAPT management in patients with HBR.

## Introduction

Cardiovascular diseases caused 6.2 million deaths among individuals aged 30-70 years, with low- and middle-income countries accounting for 80% of the burden [1]. In India, acute coronary syndrome (ACS) presents a significant health challenge, with in-hospital mortality rates ranging from 5% to 15% [2,3]. Mortality from coronary artery disease (CAD) in Asian Indians is 20% to 50% higher than that in other populations, often due to risk factors such as hypertension, diabetes, dyslipidaemia, smoking, and obesity [3]. In India, the burden of ischemic heart disease (IHD), including ACS and CAD, has increased exponentially, coinciding with the expanded use of coronary catheterization and percutaneous coronary intervention (PCI). The number of PCI procedures has increased annually by 3.7%, 13.1%, 12.6%, and 12.9% in 2017, 2018, 2019, and 2021, respectively. India currently faces the challenge of ensuring that invasive cardiovascular procedures are performed safely and efficiently [4]. Therefore, adopting a heart team approach is essential for identifying risks and requires a comprehensive evaluation due to the mortality risk associated with complex PCI procedures [5].

Dual antiplatelet therapy (DAPT) with a purinergic receptor P2Y12 (G-protein coupled) inhibitor is the standard of care for patients with ACS before and after post-PCI [6]. The American College of Cardiology/American Heart Association (ACC/AHA) and European Society of Cardiology (ESC) guidelines for managing ACS recommend six months of DAPT for stable CAD and 12 months for ACS [7,8]. However, antiplatelet therapy is not without risks. Although it reduces the risk of thrombotic events, it increases the risk of bleeding. Bleeding is the most common non-cardiac complication in patients undergoing PCI and is associated with a higher risk of death, myocardial infarction (MI), stroke, and extended hospital stay [9,10]. Guidelines recommend evaluating each patient using risk scores to minimize bleeding complications, especially in those with high bleeding risk (HBR) [7-10]. However, HBR remains poorly defined, and its criteria vary across studies.

The Academic Research Consortium (ARC) for HBR proposed a definition with 17 major and minor criteria to standardize HBR. The ARC-HBR group recognized that defining bleeding risk is essential for understanding the risks and benefits in HBR patients pre- and post-PCI. Managing the risk of bleeding must be balanced with the assessment of thrombotic risk [11]. A Korean study found that patients with HBR who underwent PCI had a higher risk of bleeding, ischemic events, and mortality than those without HBR. Using the ARC-HBR criteria, these patients exhibited increased long-term risks compared with non-HBR patients [12]. The safety of transitioning from dual DAPT to single antiplatelet therapy (SAPT) in patients with HBR remains unclear. The Indian interventional cardiologist’s perspective on de-escalation from dual to single antiplatelet therapy (INDEPTH) study by Mohan et al. found that most patients with CAD received DAPT post-PCI, with clopidogrel used in 50%-75% of SAPT-eligible patients [13]. The ACC/AHA and ESC guidelines recommend DAPT with clopidogrel and aspirin for six months post-PCI, followed by SAPT, to lower MACE and bleeding risks [7,8].

Managing Indian ACS patients with HBR post-PCI is challenging owing to the heterogeneity of HBR definitions, diversity of methods for assessing bleeding risk, limited access to simple bleeding risk tools, and the lack of region-specific guidelines for DAPT de-escalation. Additionally, India has a high prevalence of diabetes, chronic kidney disease (CKD), anemia, and tropical illnesses, such as dengue and malaria, which elevate bleeding risks and complicate ACS management [14-18]. Thrombocytopenia (TCP), a common feature of these tropical diseases, is characterized by significantly reduced platelet counts, sometimes as low as 5,000/mm³, and may be accompanied by a packed cell volume above 50%. It is also associated with HBR. It is recommended to continue DAPT with platelet monitoring in patients with HBR and DES, switch from prasugrel or ticagrelor to clopidogrel, and maintain aspirin at 75-100 mg. If the platelet counts fall below 50000/mm³, DAPT should be discontinued or switched to SAPT [18]. As there is no standardized approach for bleeding risk assessment in HBR post-PCI, this study aimed to gather insights from Indian interventional cardiologists, who are the primary decision-makers for peri- and post-PCI DAPT duration and routinely manage these high-risk patients, on how they evaluate bleeding risk factors, their need for simple assessment tools, and their practical strategies for optimizing DAPT therapy in HBR patients post-PCI.

## Materials and methods

The INDEPTH-HBR was a cross-sectional observational study conducted between June 2024 and July 2024. This study aimed to gather insights from Indian interventional cardiologists on de-escalating DAPT to SAPT in patients with post-PCI HBR. A total of 400 cardiologists were invited to participate in the study, using a convenience sampling approach. This study recruited academic interventional cardiologists from various metropolitan and non-metropolitan (Tiers I-IV cities) healthcare settings in India. The selection was based on 15 years of clinical expertise in managing patients with ACS and experience with DAPT regimens, particularly for post-PCI HBR patients. Convenience sampling was employed because interventional cardiologists are often engaged in critical care and emergency duties, which makes structured recruitment challenging. This study adhered to the ethical principles outlined in the Declaration of Helsinki and ensured data confidentiality and compliance with the relevant privacy regulations. As the study did not involve the collection of patient data or interventions, ethical approval was not mandatory according to the Indian Council of Medical Research, 2017 [19]. Voluntary responses to the survey were considered consent to participate. All responses were anonymized to ensure confidentiality.

Questionnaire development and study conduct

A structured questionnaire was used to collect the data. The questionnaire was developed based on an extensive literature review focusing on recent global and Indian clinical evidence and guidelines for DAPT de-escalation in HBR. It evaluated the current practices for transitioning from DAPT to SAPT in various clinical scenarios, factors influencing the decision to de-escalate therapy, challenges and barriers to implementing SAPT in high-risk patients, and preferences for antiplatelet and P2Y12 agents after de-escalation therapy. A pilot study with four interventional cardiologists with 20 years of experience in managing patients with HBR assessed the clarity, relevance, and thoroughness of the questionnaire items. The questionnaire was revised based on feedback and suggestions. The final questionnaire consisted of 17 closed-ended questions or statements addressing various aspects of the current practices for transitioning from DAPT to SAPT in real-world settings. The final survey was distributed via an online link sent to registered e-mail addresses. It included a URL that directed recipients to an online version of the questionnaire that was created and hosted on Google Forms. Responses were collected as multiple-choice or option-based answers, enabling quantitative analysis of practice patterns.

Data analysis

The completed survey forms from 375 of the 400 interventional cardiologists were transferred to Microsoft Excel (Microsoft Corp., Redmond, WA, US) for the analysis. SPSS Version 29 (IBM Corp., Armonk, NY, USA) was used for data analysis. Descriptive statistics, such as frequencies and percentages, were used to summarize the distribution of the responses to the survey questions. Cross-tabulations and Chi-square tests were used to explore the associations between variables. Statistical significance was set at p<0.05, and a 95% confidence interval was used to interpret the findings.

## Results

Clinical practices in managing high bleeding risk patients after percutaneous coronary intervention

Of the 375 cardiologists, 193 (51.47%) reported an HBR prevalence of less than 10% and 28 (7.47%) reported a prevalence of 21%-30%. Nearly 250 (66.67%) cardiologists reported that gastrointestinal (GI) bleeding was the most common type of bleeding observed in post-PCI patients receiving DAPT, and 171 (45.60%) reported that <5% of patients experienced actionable bleeding after DAPT. Most of the 276 interventional cardiologists (73.60%) supported the simplification of bleeding risk scores. Patients aged ≥70 years were more prone to HBR, as reported by 201 (53.60%). When defining anemia in HBR risk scoring, 174 (46.40%) preferred hemoglobin <11 g/dL, 172 (45.87%) preferred hemoglobin <10 g/dL, and 162 (43.20%) recommended a platelet count of <100×10^9^/L. Post-tropical disease management involved restarting therapy immediately for 151 (40.27%) cardiologists, whereas 197 (52.53%) felt that <10% of post-PCI patients were eligible for SAPT. The most common antiplatelet agent was clopidogrel (75 mg), which was chosen by 128 cardiologists (34.13%) (Table 1). All survey findings were statistically significant (p<0.001), with confidence intervals indicating high precision and consistency in cardiologists’ responses.

**Table 1:** Clinical insights and practice patterns reported by Indian cardiologists regarding the prevalence, management, and challenges of HBR patients post-PCI PCI: percutaneous coronary intervention; DAPT: dual antiplatelet therapy; CKD: chronic kidney disease; HBR: high bleeding risk, SAPT: single antiplatelet therapy; CI: confidence interval, n: number of participants; GI: gastrointestinal

Questions	Options	HCP Response n (%)	P-value	95% CI
What is the prevalence of HBR in your clinical practice in post-PCI patients?	<10%	193 (51.47%)	<0.001	1.93 to 2.15
	10%-20%	153 (40.80%)		
	21%-30%	28 (7.47%)		
	>30%	1 (0.27%)		
Which is the most common type of bleeding observed in post-PCI patients who are on DAPT? (Most common to least common)	Gastrointestinal bleeding	250 (66.67%)	<0.001	1.48 to 1.66
	Subcutaneous bleeding	56 (14.93%)		
	Intracranial bleeding	50 (13.33%)		
	Genitourinary bleeding	19 (5.07%)		
What is the percentage of patients who report actionable bleeding after DAPT?	<5%	171 (45.60%)	<0.001	1.62 to 1.76
	5%–10%	151 (40.27%)		
	10%–20%	52 (13.87%)		
	>20%	1 (0.27%)		
What is the percentage of patients who report non-actionable bleeding after DAPT?	5%–10%	151 (40.27%)	<0.001	2.21 to 2.39
	10%–20%	110 (29.33%)		
	<5%	75 (20.00%)		
	>20%	39 (10.40%)		
Do you feel the bleeding risk score for estimating HBR patients should be simplified?	Simplified	276 (73.60%)	<0.001	2.63 to 2.74
	Not necessary	81 (21.60%)		
	No comment	18 (4.80%)		
In the Indian setup which age group is more prone to high bleeding risk?	>70 years	201 (53.60%)	<0.001	2.02 to 2.15
	>/=80 Years	103 (27.47%)		
	>/=60 Years	71 (18.93%)		
While defining cut-off for anemia in bleeding risk score for Indian setup; what can be the defining hemoglobin levels?	<11	174 (46.40%)	<0.001	2.32 to 2.44
	<10	172 (45.87%)		
	<12	29 (7.73%)		
What should be the cut-off platelet count to label a patient as HBR?	<100×10^9^/L	162 (43.20%)	<0.001	1.93 to 2.14
	(50–99)×10^9^/L	96 (25.60%)		
	(100–149)×10^9^/L	77 (20.53%)		
	<50×10^9^/L	40 (10.67%)		
In such type of patients (who have recent history of tropical disease), will you consider it as a risk factor for HBR and de-escalate from DAPT?	Sometimes	163 (43.47%)	<0.001	3.17 to 3.32
	Yes	154 (41.07%)		
	No	53 (14.13%)		
	No comment	5 (1.33%)		
How often do you consider shortening the duration of DAPT due to spontaneous bleeding?	Sometimes	190 (50.67%)	<0.001	2.02 to 2.18
	Selected patients	85 (22.67%)		
	Frequently	83 (22.13%)		
	Rarely	17 (4.53%)		
How often do you stop antithrombotic agents due to GI bleeding?	Sometimes	214 (57.07%)	<0.001	2.11 to 2.26
	Selected patients	94 (25.07%)		
	Frequently	53 (14.3%)		
	Rarely	14 (3.73%)		
When will you restart antiplatelet therapy after an episode of tropical disease?	Immediately	151 (40.27%)	<0.001	1.98 to 2.16
	After 1 week	125 (33.33%)		
	Continue during the tropical disease episode	99 (26.40%)		
What percentage of HBR patients do you feel are eligible for SAPT immediately post PCI?	<10%	197 (52.53%)	<0.001	1.90 to 2.06
	<5%	103 (27.47%)		
	<20%	54 (14.40%)		
	<30%	21 (5.60%)		
Post PCI in HBR patients which is your preferred choice of antiplatelet therapy following short or ultra-short DAPT?	Clopidogrel 75 mg	128 (34.13%)	<0.001	3.03 to 3.37
	Aspirin 75 mg	107 (28.53%)		
	Ticagrelor 90 mg	63 (16.80%)		
	Ticagrelor 60mg	44 (11.73%)		
	Aspirin 150 mg	32 (8.53%)		
	Prasugrel 5 mg	1 (0.27%)		
When will you restart antiplatelet therapy after a bleeding episode?	After 2-4 weeks	154 (41.07%)	<0.001	2.43 to 2.60
	After 1 week	143 (38.13%)		
	After 1 month	39 (10.40%)		
	No Comment	39 (10.40%)		

Assessment of high bleeding risk based on risk factors

Of the 375 cardiologists, 326 (86.9%) emphasized frailty, 314 (83.7%) emphasized severe or CKD stage 3, and 324 (86.4%) emphasized previous spontaneous intracerebral hemorrhage (ICH) as a highly important risk factor for HBR assessment. Patients aged ≥60 years and with a body weight <60 kg were of high importance to 285 (76.00%) and 293 (78.13%) cardiologists, respectively. Nondeferrable major surgery with DAPT was considered moderately important in 249 (66.40%) patients. Hemoglobin level <11 g/dL and steroids for any disease were given very low importance by 213 (56.80%) and 202 (53.87%) cardiologists, respectively (Table 2). Each assessed HBR risk factor showed a highly significant association (p<0.001), reflecting a strong consensus among the respondents regarding their clinical relevance.

**Table 2:** HBR risk assessment based on risk factors PCI: percutaneous coronary intervention; DAPT: dual antiplatelet therapy; CKD: chronic kidney disease; CI: confidence interval; NSAIDS: non-steroidal anti-inflammatory drugs, n: number of participants

Criteria	Significant n (%)	Non-significant n (%)	P-value	95% CI
1) Age ≥60 years	285 (76.00%)	90 (24.00%)	<0.001	1.72 to 1.80
2) Frailty	326 (86.93%)	49 (13.07%)	<0.001	1.84 to 1.90
3) Body weight <60kg	293 (78.13%)	82 (21.87%)	<0.001	1.74 to 1.82
4) Use of long-term oral anticoagulation	314 (83.73%)	61 (16.27%)	<0.001	1.80 to 1.87
5) Severe or CKD stage 3	319 (85.07%)	56 (14.93%)	<0.001	1.81 to 1.89
6) Haemoglobin <11g/dL	213 (56.80%)	162 (43.20%)	<0.001	1.52 to 1.62
7) Spontaneous bleeding requiring hospitalization or transfusion in the past 6 months or at any time, if recurrent	311 (82.93%)	64 (17.07%)	<0.001	1.79 to 1.87
8) Spontaneous bleeding requiring hospitalization or transfusion within the past 12 months not meeting the major criteria	291 (77.60%)	84 (22.40%)	<0.001	1.73 to 1.82
9) Moderate or severe baseline thrombocytopenia (platelet count <100×10^9^/L)	311 (82.93%)	64 (17.07%)	<0.001	1.79 to 1.87
10) Chronic bleeding diathesis	309 (82.40%)	66 (17.60%)	<0.001	1.79 to 1.86
11) Liver cirrhosis with portal hypertension	324 (86.40%)	51 (13.60%)	<0.001	1.83 to 1.90
12) Long-term use of oral NSAIDs	306 (81.60%)	69 (18.40%)	<0.001	1.78 to 1.86
13) Active malignancy (excluding nonmelanoma skin cancer) within the past 12 months	289 (77.07%)	86 (22.93%)	<0.001	1.73 to 1.81
14) Previous spontaneous ICH (at any time)	309 (82.40%)	66 (17.60%)	<0.001	1.79 to 1.86
15) Non-deferrable major surgery on DAPT	249 (66.40%)	126 (33.60%)	<0.001	1.62 to 1.71
16) Recent major surgery or major trauma within 30 days before PCI	272 (72.50%)	103 (27.50%)	<0.001	1.68 to 1.77
17) Prior bleed before hospitalization	274 (73.07%)	101 (26.93%)	<0.001	1.69 to 1.78
18) On steroids for any disease	202 (53.87%)	173 (46.13%)	<0.001	1.49 to 1.59

Cardiologists' preferences for P2Y12 inhibitors

**Figure 1:**
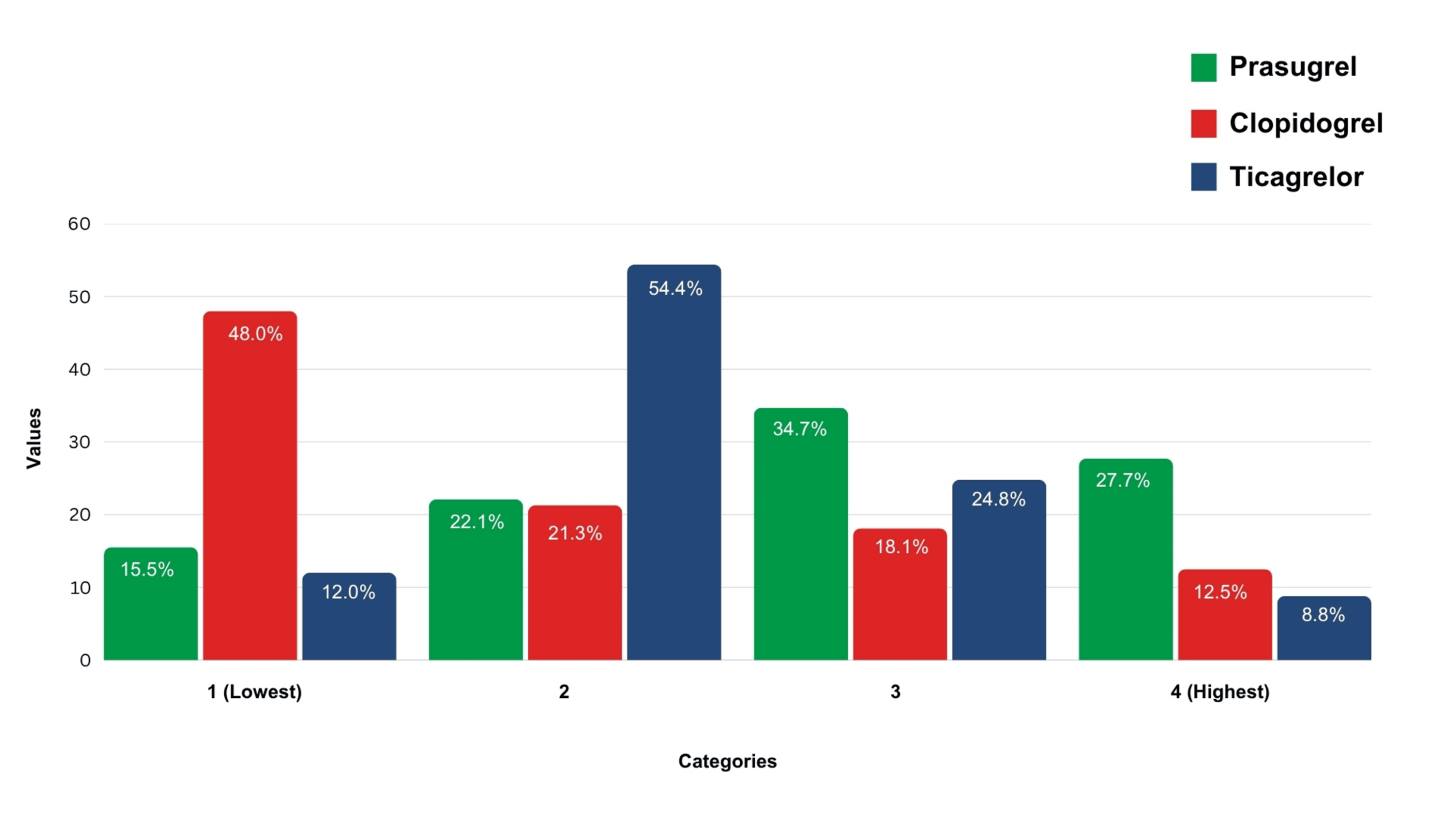
Interventional cardiologists’ preferences for P2Y12 inhibitors (drugs with high tendency of bleeding risk, 1-lowest, 4-highest) (clopidogrel, prasugrel, and ticagrelor)

**Figure 2:**
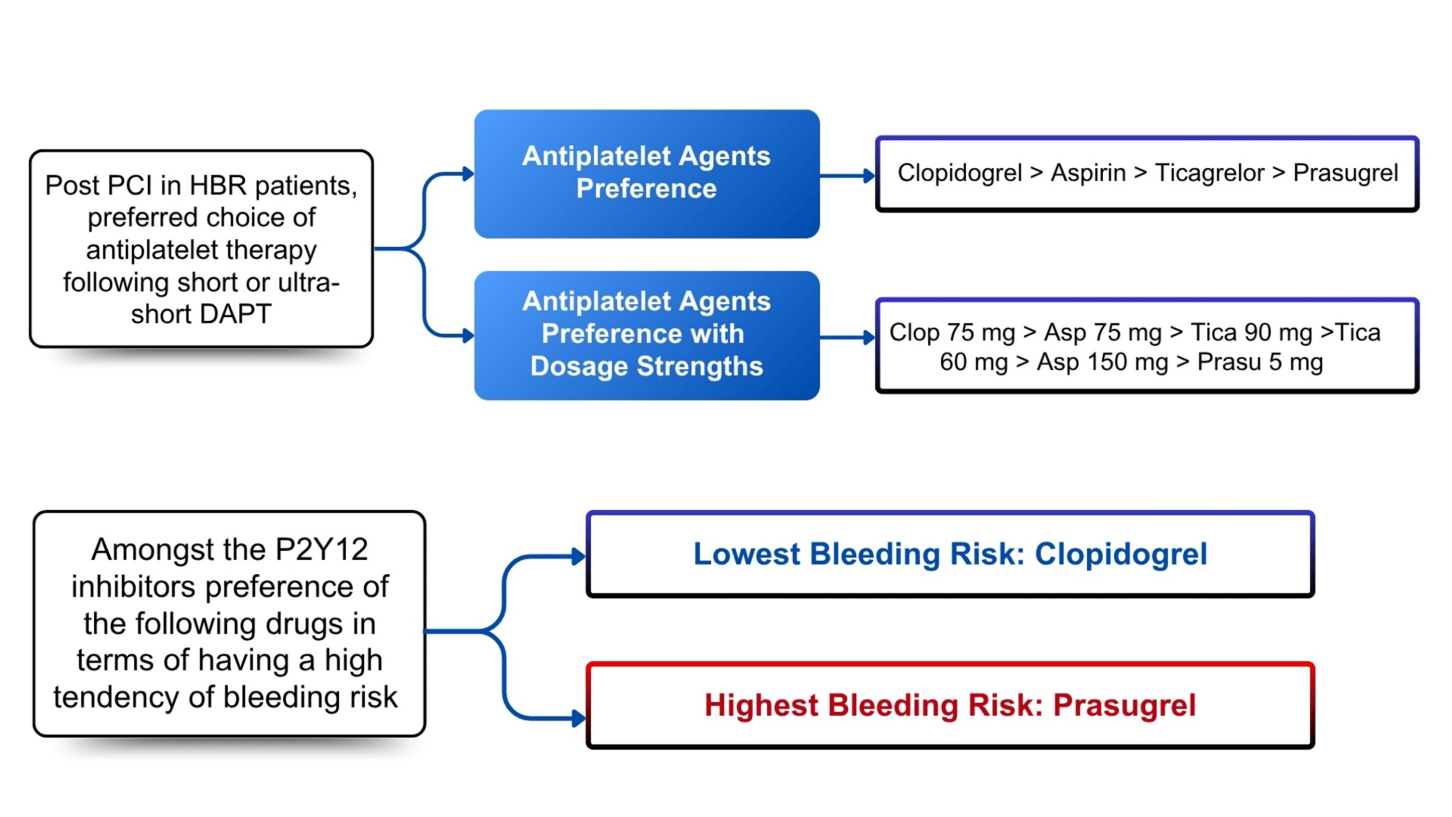
Interventional cardiologists’ preferences for P2Y12 inhibitors post-PCI in HBR patients Clop: clopidogrel, Asp: aspirin, Tica: ticagrelor, Prasu: prasugrel; HBR: high bleeding risk; PCI: percutaneous coronary intervention

Based on perceived bleeding risk, 179 cardiologists (48.00%) rated clopidogrel as the least likely to cause bleeding. It was predominantly chosen for patients with HBR transitioning from DAPT to SAPT, with 80 (21.33%) cardiologists rating it 2. Nearly 63.00% of cardiologists considered prasugrel to have a HBR, and 66.40% considered ticagrelor to have a moderate bleeding risk. Most cardiologists (70.00%) considered clopidogrel to have the lowest risk (Figures 1, 2).

High bleeding risk factor categorization

**Figure 3:**
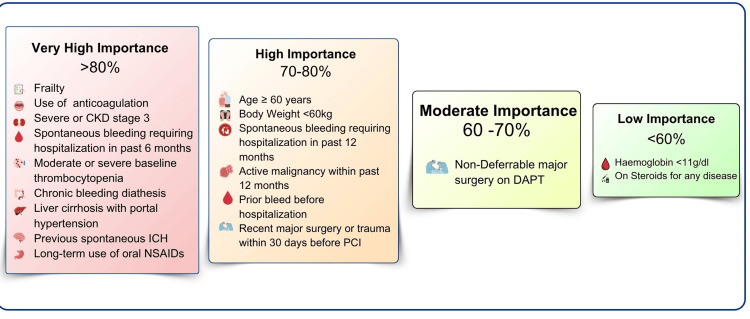
HBR factor categorization based on Indian cardiologist perspectives HBR: high bleeding risk; DAPT: dual antiplatelet therapy; CKD: chronic kidney disease; ICH: intracerebral hemorrhage; PCI: percutaneous coronary intervention; NSAIDs: non-steroidal anti-inflammatory drugs

Factors such as frailty, CKD Stage 3 or severe, liver cirrhosis with portal hypertension, long-term oral anticoagulant use, and moderate or severe baseline TCP were given high importance (>80%). Age ≥ 60 years, body weight <60 kg, and spontaneous bleeding requiring hospitalization in the past 12 months were given high importance (70%-80%). Non-deferrable major surgery during DAPT was assigned moderate importance (60%-70%). Hemoglobin <11 g/dL and steroids for any disease were given low importance (<60%) by the surveyed interventional cardiologists (Figure 3).

All criteria used to categorize patients are shown in Figure 3, based on their perceived importance to Indian cardiologists in influencing antiplatelet therapy decisions post-PCI. Factors considered of very high importance (>80%) include frailty, CKD stage 3 or higher, liver cirrhosis with portal hypertension, long-term oral anticoagulation, moderate or severe baseline TCP, chronic bleeding diathesis, long-term NSAID use, and a history of spontaneous ICH. Factors of high importance (70%-80%) include age ≥60 years, body weight <60 kg, spontaneous bleeding requiring hospitalization in the past 12 months, active malignancy within the past 12 months, bleeding prior to hospitalization, and recent major surgery or trauma within 30 days before PCI. Moderate importance factors (60%-70%) include the need for non-deferrable major surgery during DAPT. Low importance factors (<60%) include hemoglobin <11 g/dL and steroid use for any disease.

## Discussion

The findings of the current INDEPTH survey among eminent Indian interventional cardiologists provide valuable insights into real-world perspectives for managing patients with HBR post-PCI. GI bleeding has emerged as the most common complication associated with DAPT, with actionable bleeding reported more frequently in a small subset of patients. Cardiologists have reached a strong consensus on simplifying the bleeding risk scores to improve their applicability in clinical practice. Frailty, CKD, liver cirrhosis, TCP, and long-term oral anticoagulant therapy were the most critical risk factors influencing antiplatelet therapy decisions. Clopidogrel was the preferred antiplatelet agent for de-escalation to SAPT due to its perceived lower risk of bleeding, while ticagrelor and prasugrel were reserved for specific cases. This study also highlighted the challenges of restarting therapy after bleeding or in scenarios such as recovery from tropical diseases, emphasizing the need for individualized management strategies.

In the current survey, more than half of the interventional cardiologists reported an HBR prevalence of less than 10% in post-PCI patients, which is consistent with the published literature. A meta-analysis by Moghadam et al. reported that the incidence of bleeding after PCI was 4.4%, while Chang et al. showed that the bleeding prevalence varied widely, ranging from 1% to 10% in clinical trials and 2.8% to 11% in real-world studies, with a significant impact on mortality [20]. The ARC-HBR criteria are well-validated, classifying approximately 50% of patients as having an HBR, who subsequently experience higher post-PCI bleeding rates [21]. Indian interventional cardiologists identified CKD stage 3 or higher, ICH, TCP, and bleeding diathesis as the major diagnostic criteria. Frailty and low body weight (<60 kg) were deemed significant minor ARC-HBR criteria owing to the prevalence of malnutrition in Indian patients. Liver cirrhosis is prioritized owing to the prevalence of alcohol-related diseases and viral hepatitis. Long-term NSAID use is critical to reduce the risk of GI bleeding. Spontaneous bleeding and recent surgery were prioritized because of the delayed access to healthcare. Anemia has received less attention owing to its widespread prevalence. A few interventional cardiologists in the current survey applied stricter anemia cut-offs (<10 g/dL) and platelet thresholds (<(75-80)×10^9^/L) than the ARC-HBR definitions (anemia <11 g/dL, platelet count <100×10^9^/L). According to the ARC-HBR, patients are at HBR if they meet one major or two minor criteria, including severe CKD, prior ICH, TCP, bleeding diathesis, and active malignancy [11,22]. Although the survey responses partially align with the ARC-HBR criteria, this survey highlights the need to consider India-specific factors in HBR assessments in post-PCI patients.

In the current survey, 48% of cardiologists rated clopidogrel as least likely to cause bleeding. It was mainly selected for patients with HBR who transitioned from DAPT to SAPT after PCI. Similarly, Cayla et al. discussed de-escalation from potent P2Y12 inhibitors to clopidogrel as an alternative to short DAPT duration in patients with HBR. P2Y12 inhibitor de-escalation strategies have been explored to reduce the risk of bleeding in patients with HBR-ACS undergoing PCI [23]. A meta-analysis by Kang et al. demonstrated that de-escalation of DAPT compared with standard DAPT led to a reduction in ischemic and bleeding endpoints in patients with ACS who underwent PCI [24]. The TALOS-AMI sub-study found that de-escalation from ticagrelor to clopidogrel reduced the composite endpoints of cardiovascular death, MI, stroke, and bleeding in the HBR subgroup. In contrast, the non-HBR subgroup showed no significant reduction in bleeding [25,26]. The MASTER DAPT trial indicated that shortened DAPT (1-3 months) reduces the risk of bleeding in patients with HBR. De-escalation is preferred for patients with high bleeding and ischemic risks, particularly in complex PCI cases [27]. However, further trials are required to confirm the efficacy of de-escalation therapy in high-risk cohorts in India.

In the current survey, 73.60% of cardiologists supported simplifying the bleeding risk scores. A systematic review by Brinza et al. found that multiple bleeding risk scores indicate the need for an accurate model to optimize DAPT duration and reduce ischemic risk without increasing the risk of bleeding. Each score has benefits and limitations based on its development cohorts and applies to specific patients and clinical settings. The ARC-HBR and BleeMACS scores, validated in large cohorts, offer promising alternatives to PRECISE-DAPT. However, more prospective studies are needed to establish a relationship between the outcomes and the use of these clinical tools [28]. Ray et al. evaluated the PRECISE-DAPT score in Indian patients to guide the initiation of DAPT in patients with CCS [29]. However, the broader validation of both the PRECISE-DAPT and ARC-HBR scores in Indian and Asian populations remains limited. Current survey data indicate that factors such as baseline hemoglobin levels and chronic steroid use, which are highly important in ARC-HBR scores, are perceived as having low importance in Indian clinical practice. Thus, there is a significant opportunity to prospectively evaluate these tools in Indian patients with HBR-ACS to ensure that risk-prediction models are optimized for regional patient profiles and clinical decision making.

In the current survey, 40.27% of cardiologists resumed antiplatelet therapy for post-tropical disease management. Ehelepola et al. reported severe TCP, platelet dysfunction, and impaired clotting in dengue hemorrhagic fever (DHF). Continuing DAPT increases the risk of hemorrhage, whereas withholding antiplatelet therapy in patients with coronary stents can cause thrombosis, MI, and death. There are no guidelines for managing DHF in patients with a stent. This need is urgent, as 3.9 billion people are at risk of dengue, and coronary stenting is the standard treatment. Developing countries account for 82% of disability-adjusted life years (DALYs) due to coronary disease, creating a risk of dengue in dengue-endemic areas. Guidelines remain challenging because of the varying thrombosis risks and dengue spectrum. Until evidence-based guidelines are available, management should be tailored to individual risks and DHF phases [30].

Implications for Indian practice

The findings of this study are partially aligned with global efforts, such as the ARC-HBR and PRECISE-HBR scores, which aim to improve bleeding risk stratification by integrating clinical and laboratory parameters in the Indian context. However, these results emphasize the need for regional adaptations to address the factors emphasized by Indian cardiologists, including frailty, low body weight, liver cirrhosis, and long-term NSAID use, which have not been prioritized in the previous studies. Additionally, tropical diseases such as dengue and malaria are known to cause TCP and platelet dysfunction, complicating bleeding risk management and antiplatelet therapy in India. This study is the first to categorize HBR risk factors based on their perceived clinical importance by Indian interventional cardiologists as very high (>80%), high (70%-80%), moderate (60%-70%), and low (<60%). Factors such as frailty, severe CKD, and liver cirrhosis with portal hypertension were rated as being of very highly important, indicating their strong influence on post-PCI antiplatelet therapy decisions. This structured prioritization may provide a practical framework to assist Indian cardiologists in making informed decisions regarding DAPT optimization in patients with HBR post-PCI.

Strengths and limitations

The strength of this study lies in its ability to capture clinical insights from 375 interventional cardiologists across India regarding the real-world challenges and practices in managing patients with HBR after PCI. This is among the first studies to explicitly explore the perceptions of de-escalation from DAPT to SAPT in HBR patients, an increasingly important area globally, with a notable lack of data from India. Considering India’s unique patient characteristics (e.g., higher rates of CKD, anemia, and liver disease), these findings offer regionally tailored insights into antiplatelet therapy decisions and address critically unmet needs. Expert perspectives on current practice patterns can serve as a practical guide for Indian cardiologists in real-world settings; however, they should be viewed with caution, as they reflect perceived rather than actual clinical behavior.

Despite these strengths, this study has certain limitations. This study employed convenience sampling to recruit interventional cardiologists, which may limit its generalizability to all Indian clinicians, particularly those practicing in nonurban or resource-limited settings. As interventional cardiologists are frequently engaged in critical care and emergency procedures, convenience sampling was employed to ensure adequate participation within practical time constraints. However, this approach may limit representativeness, as non-interventional cardiologists, primary care physicians, and other providers involved in HBR patient management were not included, thereby limiting the generalizability of the results to interventional cardiologists. The survey relied on self-reported responses, which may have introduced a recall bias or subjective interpretations of clinical practice. As this was a perception-based survey, a formal psychometric validation was not conducted. However, content and face validity were ensured by a panel of ten subject-matter experts who reviewed and approved all the questionnaire items before the survey was disseminated. This survey focused on expert consensus and clinician perspectives, and often overlooked patient-level clinical outcomes. However, this was beyond the scope of the present study. To strengthen the study findings, we recommend conducting longitudinal studies that assess the impact of de-escalation strategies on clinical outcomes, such as bleeding events, ischemic complications, or mortality, in ACS patients with HBR across diverse HBR patient groups, thereby adding significant clinical value to the study.

## Conclusions

This survey provides preliminary, non-generalizable insights into the perspectives of Indian interventional cardiologists in managing patients after post-PCI and HBR, focusing on de-escalating DAPT to SAPT. Key findings identified frailty, CKD, liver cirrhosis with portal hypertension, and spontaneous bleeding as the most critical risk factors for HBR management in India. The results demonstrated a strong preference for clopidogrel as the antiplatelet agent of choice for de-escalation in patients with HBR because of its favorable safety profile. These findings emphasize the perceived need among Indian interventional cardiologists for simplified risk-scoring tools to support clinical decision-making and promote a more personalized and practical approach to managing patients with HBR, as perceived by prominent interventional cardiologists. However, future research should examine patient-level outcomes and longitudinal data to confirm these findings and strengthen evidence-based practices.

## References

[1] Roth GA, Mensah GA, Johnson CO (2020). Global burden of cardiovascular diseases and risk factors, 1990-2019: update from the GBD 2019 study. J Am Coll Cardiol.

[2] Gupta R, Gaur K (2020). Epidemiology of acute coronary syndromes in India. Acute Coronary Syndromes.

[3] Sanchis-Gomar F, Perez-Quilis C, Leischik R, Lucia A (2016). Epidemiology of coronary heart disease and acute coronary syndrome. Ann Transl Med.

[4] Bhanu D, Gupta I, Kumar U, Rayapati V, Saunik S, Gedam P (2023). E-20| appropriateness of percutaneous coronary intervention under Ayushman Bharat Pradhan Mantri Jan Arogya Yojana (AB-PMJAY). J Soc Cardiovasc Angiogr Interv.

[5] Gogineni VS, Shah KB (2024). High-risk percutaneous coronary intervention: challenges and considerations. Cardiovasc Innov Appl.

[6] Hong SJ, Kim BK (2025). Potent P2y(12) inhibitor monotherapy for acute coronary syndrome. Circ J.

[7] Byrne RA, Rossello X, Coughlan JJ (2023). 2023 ESC Guidelines for the management of acute coronary syndromes: developed by the task force on the management of acute coronary syndromes of the European Society of Cardiology (ESC). Eur Heart J.

[8] Rao SV, O'Donoghue ML, Ruel M (2025). 2025 ACC/AHA/ACEP/NAEMSP/SCAI guideline for the management of patients with acute coronary syndromes: a report of the American College of Cardiology/American Heart Association Joint Committee on clinical practice guidelines. J Am Coll Cardiol.

[9] Angiolillo DJ, Galli M, Collet JP, Kastrati A, O'Donoghue ML (2022). Antiplatelet therapy after percutaneous coronary intervention. EuroIntervent.

[10] Ismail N, Jordan KP, Rao S, Kinnaird T, Potts J, Kadam UT, Mamas MA (2019). Incidence and prognostic impact of post discharge bleeding post acute coronary syndrome within an outpatient setting: a systematic review. BMJ Open.

[11] Urban P, Mehran R, Colleran R (2019). Defining high bleeding risk in patients undergoing percutaneous coronary intervention. Circ.

[12] Kang J, Yun J, Park KW (2024). Long-term outcomes of high bleeding risk patients undergoing percutaneous coronary intervention: a Korean nationwide registry. Eur Heart J.

[13] Mohan JC, Singhal A, Oomman A, Ray S, Shah VT, Nath B, Bachani D (2024). Indian perspective on de-escalation from dual antiplatelet therapy to single antiplatelet therapy study: a knowledge, attitude, and practice study among Indian interventional cardiologists. J Assoc Physicians India.

[14] Alfaddagh A, Khraishah H, Romeo GR (2024). Cardiovascular outcomes among patients with acute coronary syndromes and diabetes: results from ACS Quik trial in India. Glob Heart.

[15] (2025). Cardiological Society of India, Guidelines: CCSI. Position statement on diagnosis and management of acute coronary syndrome in patients with chronic kidney disease. Accessed on 21st August. https://www.csi.org.in/guidelines.

[16] Bhavanadhar P, Srinivasan VR, Oruganti SS, Adiraju KP (2016). A prospective study on prevalence and causes of anaemia in patients with acute coronary syndrome. J Clin Diagn Res.

[17] Dutta O, Prasanth A, Kumari A, Akanksha K, Deeba F, Salam N (2023). Burden of dengue, leishmaniasis and lymphatic filariasis in India and its states from 1990-2019: Analysis from the Global Burden of Disease study (GBD 2019). PLoS One.

[18] Bhatia V (2017). Dengue fever, thrombocytopaenia and management issues in post-coronary stenting patients. AsiaIntervention.

[19] Mathur R, Swaminathan S (2018). National ethical guidelines for biomedical & health research involving human participants, 2017: a commentary. Indian J Med Res.

[20] Heidary Moghadam R, Mohammadi A, Salari N, Ahmed A, Shohaimi S, Mohammadi M (2024). The prevalence of bleeding after percutaneous coronary interventions: a systematic review and meta-analysis. Indian Heart J.

[21] Chang CC, Ng AK, Kogame N, Huang PH, Kim BK, van Geuns RM (2025). Decoding bleeding risks and survival in patients undergoing percutaneous coronary intervention on antiplatelet therapy. JACC Asia.

[22] Miura K, Shimada T, Ohya M (2020). Prevalence of the Academic Research Consortium for high bleeding risk criteria and prognostic value of a simplified definition. Circ J.

[23] Cayla G, Lattuca B (2023). De-escalation from potent P2Y12 inhibitors to clopidogrel: an alternative to short DAPT duration in HBR patients?. EuroIntervention.

[24] Kang J, Rizas KD, Park KW (2023). Dual antiplatelet therapy de-escalation in acute coronary syndrome: an individual patient meta-analysis. Eur Heart J.

[25] Kim MC, Ahn SG, Cho KH (2023). De-escalation from ticagrelor to clopidogrel in patients with acute myocardial infarction: the TALOS-AMI HBR substudy. EuroIntervention.

[26] Cao D, Mehran R, Dangas G (2020). Validation of the academic research consortium high bleeding risk definition in contemporary PCI patients. J Am Coll Cardiol.

[27] Valgimigli M, Frigoli E, Heg D (2021). Dual antiplatelet therapy after PCI in patients at high bleeding risk. N Engl J Med.

[28] Brinza C, Burlacu A, Tinica G, Covic A, Macovei L (2021). A systematic review on bleeding risk scores’ accuracy after percutaneous coronary interventions in acute and elective settings. Healthcare (Basel).

[29] Ray A, Najmi A, Khandelwal G, Jhaj R, Sadasivam B (2023). Usefulness of the PRECISE-DAPT score at differentiating between ticagrelor and prasugrel for dual antiplatelet therapy initiation. J Thromb Thrombolysis.

[30] Ehelepola ND, Athurupana AA, Bowatte PG, Dissanayake WP (2020). Continuation of dual antiplatelet therapy in a patient with a coronary artery stent with dengue hemorrhagic fever: a clinical conundrum. Am J Trop Med Hyg.

